# The Effects of Body-Oriented Interventions on Cancer-Related Symptoms of Women Who Survived Breast Cancer: Protocol for a Systematic Review

**DOI:** 10.2196/76858

**Published:** 2026-05-01

**Authors:** Daniela Guerreiro, Ana Cruz-Ferreira, Graça Duarte Santos, Brenda M. S. da Silva, José Marmeleira, Guida Veiga

**Affiliations:** 1 Departamento de Desporto e Saúde Escola de Saúde e Desenvolvimento Humano University of Évora Évora Portugal; 2 Comprehensive Health Research Centre (CHRC) University of Évora Évora Portugal; 3 Department of Educational and Developmental Psychology Institute of Psychology Leiden University Leiden The Netherlands

**Keywords:** mind-body, quality of life, stress, depression, relaxation, psychomotor therapy

## Abstract

**Background:**

Breast cancer is one of the most prevalent cancers worldwide, with a 5-year survival rate exceeding 90%. Despite advances in treatment, survivors frequently experience persistent cancer- and treatment-related symptoms that negatively impact their quality of life. Body-oriented interventions (BOIs) have demonstrated effectiveness in symptom management; however, systematic reviews focused exclusively on BOIs for women who survived breast cancer (WSBC) remain limited. This systematic review protocol outlines the methodology for evaluating the scientific evidence on the effects of BOIs on cancer- and treatment-related symptoms in WSBC.

**Objective:**

The aim of this study is to examine the scientific evidence on the effects of BOIs on cancer- and treatment-related symptoms, well-being, and quality of life in WSBC.

**Methods:**

This protocol follows PRISMA-P (Preferred Reporting Items for Systematic Reviews and Meta-Analyses Protocols) guidelines. We will conduct searches in 6 electronic databases: PubMed, Cochrane, Web of Science, Scopus, APA PsycNet, and Portal Regional da BVS. Studies will be considered for inclusion if they are written in English, French, Portuguese, or Spanish, with no restrictions on publication date; they consist of WSBC (aged 18 to 64 years); they are randomized controlled trials, quasi-randomized controlled trials, and pilot studies focusing on BOIs; they include a control group receiving no intervention, standard care, or a non-BOI; and the primary outcomes of interest include preintervention and postintervention measures of cancer- and treatment-related symptoms, well-being, and quality of life. The methodological quality of the studies and the risk of bias will be assessed using the PEDro scale and version 2 of the Cochrane risk-of-bias tool, respectively. The synthesis of results will be measured through the Best Evidence Synthesis. Two experienced independent reviewers will conduct study selection, data extraction, methodological quality assessment, and scientific evidence assessment. Disagreements will be resolved by a third reviewer.

**Results:**

This protocol describes the prespecified methodology for the systematic review. At the time of publication of this protocol, the corresponding full systematic review manuscript was under peer review. The outcomes will synthesize the scientific evidence on the effects of BOIs on cancer- and treatment-related symptoms in WSBC. It is anticipated that this systematic review will identify benefits and directions for future research to support the integration of BOIs tailored to this population.

**Conclusions:**

Considering that previous systematic reviews focused on the effects of BOIs in survivors of all cancer types, challenges related to study risk of bias such as heterogeneity in intervention types, study designs, and outcome measures are anticipated, potentially leading to some inconsistency and imprecision. To mitigate these issues, PRISMA guidelines will be followed, and methodological quality and best evidence strength will be rigorously assessed. This review will systematically synthesize the effects of BOIs on cancer- and treatment-related symptoms in WSBC. These findings will provide health professionals with reliable evidence and methodological guidance for further research.

**Trial Registration:**

PROSPERO CRD42023452519; https://www.crd.york.ac.uk/prospero/display_record.php?RecordID=452519

**International Registered Report Identifier (IRRID):**

DERR1-10.2196/76858

## Introduction

Breast cancer is the most common cancer among women and the second most frequently diagnosed cancer worldwide [1]. The World Cancer Research Fund International reported over 2 million new cases in 2018 [2]. Advances in early detection and treatment have significantly increased survival rates, with the 5-year survival now exceeding 90% [3]. However, the posttreatment phase remains challenging, as women who survived breast cancer (WSBC) often experience persistent physical and psychological symptoms related to cancer and its treatment that impact their well-being and quality of life (QoL) [4]. Common cancer- and treatment-related symptoms include fatigue, pain, sleep disturbances, depression, anxiety, menopausal symptoms, lymphedema, and body image alterations [4,5]. Given the complexity and impact of the cancer- and treatment-related symptoms, effective management strategies are essential to enhance well-being and QoL during the survivorship phase.

Body-oriented interventions (BOIs) encompass bodily experiences (eg, posture, muscle tone, breath, motion) and movement activities, which may function both as diagnostic tools and as therapeutic mediators [6]. By deliberately engaging sensorimotor processes to access, regulate, and integrate emotional material, BOIs foster body and emotional awareness, self-regulation, and deeper mind–body integration [5,7], providing a bottom-up pathway to emotional change. As an umbrella term, BOIs encompass psychomotricity, play, dance/movement, relaxation techniques, and exercise [7]. In oncology settings, BOIs facilitate adaptation to cancer- and treatment-related changes, in particular, to bodily alterations and emotional distress, thereby contributing to symptom management, overall health, and QoL improvement [8,9].

Despite the rising interest in BOIs to support breast cancer survival, existing systematic reviews on BOIs for WSBC are limited in scope. Prior reviews focused primarily on yoga [10] or physical activity [11], excluding other BOIs such as psychomotricity and relaxation techniques. Additionally, they often included women still undergoing active treatment [10,12,13] or mixed populations of cancer survivors [14-17]. Additionally, key cancer- and treatment-related symptoms such as pain, quality of sleep, and alterations in body schema and body image have not been included in these previous reviews. For the purposes of this review, BOIs will be initially grouped into four predefined modalities, in alignment with the data extraction framework: (1) psychomotricity, (2) dance/movement, (3) relaxation techniques, and (4) structured exercise. This preliminary categorization, informed by previous literature [6,7], aims to reduce conceptual heterogeneity and enhance comparability. However, final categorization will be confirmed after study selection and data extraction, ensuring alignment with the interventions described in the included trials.

By integrating a broad range of BOIs and outcomes, the systematic review described in this protocol will address limitations of previous studies that focused only on specific interventions or outcomes. Thus, the systematic review described in this protocol aims to examine the scientific evidence on the effects of BOIs on cancer- and treatment-related symptoms (eg, fatigue, pain, sleep quality), well-being (eg, global and domain-specific well-being measures), and QoL (eg, global and domain-specific QoL measures) in WSBC. As breast cancer survivorship trajectories are shaped not only by the biology of the disease but also by age-related physiological changes and the acute effects of ongoing treatment [18], the systematic review will include only adult women aged 18-64 years who are in at least 3 months post-treatment—thereby focusing on a stable survivorship phase [19], when long-term effects of cancer and its treatment (eg, fatigue, cognitive changes, emotional distress) become more evident and may require supportive interventions [18]. Considering previous research, it is anticipated that BOIs will have benefits for the health, well-being, and QoL of WSBC. Furthermore, the review is expected to identify critical evidence gaps and methodological considerations, thereby informing future research and guiding the optimized integration of BOIs for this population.

## Methods

### Analysis Plan

**Figure 1:**
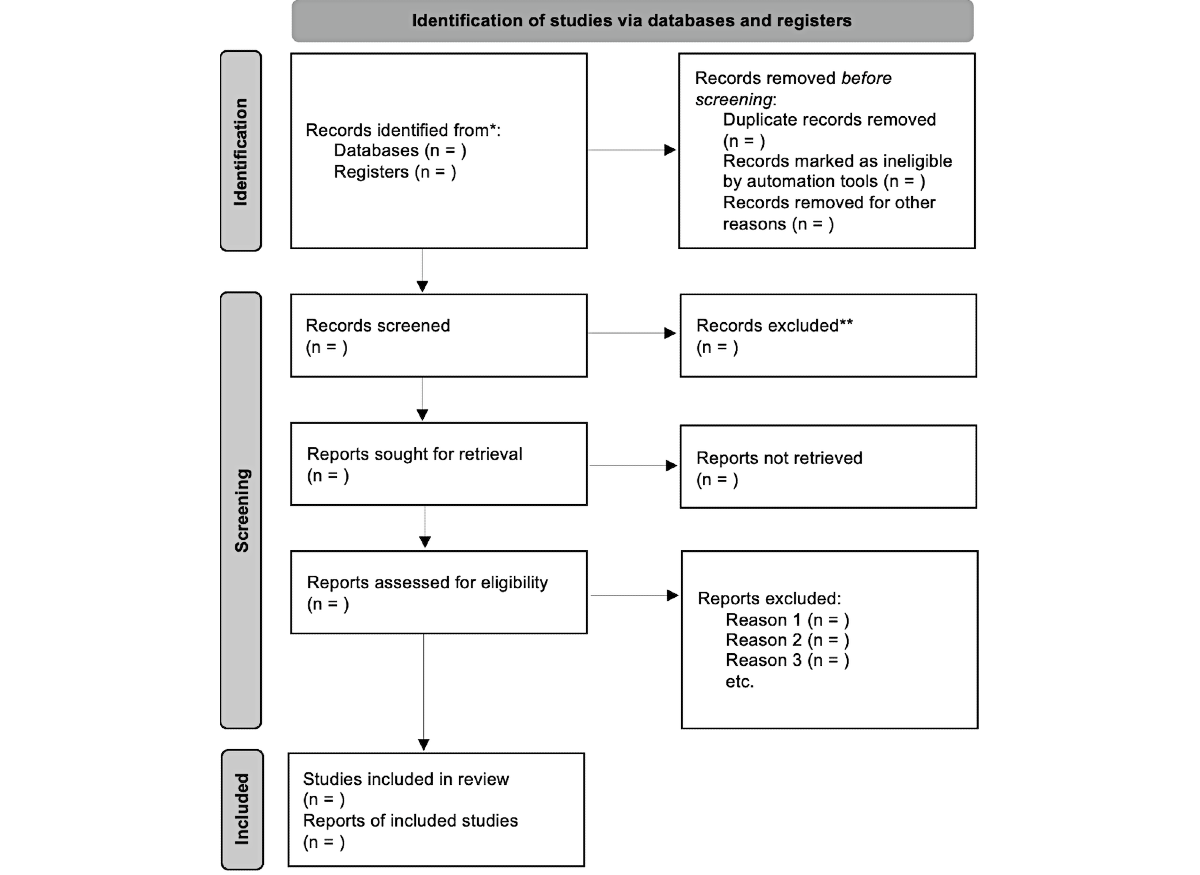
PRISMA (Preferred Reporting Items for Systematic Reviews and Meta-Analyses) flow diagram.

This protocol will be developed in accordance with the PRISMA-P (Preferred Reporting Items for Systematic Reviews and Meta-Analyses Protocols) guidelines [20] (Multimedia Appendix 1), and the systematic review will follow the PRISMA guidelines [21]. The PRISMA framework provides a structured approach to selecting studies, which will be visualized using a flowchart (Figure 1) to guide the review. Similar approaches appear in recently published systematic review protocols [22-24].

### Eligibility Criteria

This review follows the Population, Intervention, Comparison, Outcome, and Study design (PICOS) framework [25] to ensure systematic and transparent eligibility criteria (Table 1). Studies will be considered for inclusion if they were written in English, French, Portuguese, or Spanish, with no restrictions on publication date. The following inclusion criteria must be met.

**Table 1:** Population, Intervention, Comparison, Outcome, and Study design (PICOS) criteria for study selection.

PICOS element	Description
Population (P)	Women who survived breast cancer (age ≥18 to ≤64 years)
Intervention (I)	Body-oriented interventions
Comparator (C)	Control group receiving No intervention Standard care A non–body-oriented intervention
Outcome (O)	Cancer- and treatment-related symptoms Well-being Quality of life
Study type (S)	Randomized controlled trials Quasi-randomized controlled trials Pilot studies

### PICOS Framework

#### Population (P)

Women (aged 18 to 64 years) diagnosed with breast cancer who have completed primary cancer treatment (surgery, radiotherapy or/and chemotherapy) at least 3 months before study enrollment will be included. Women undergoing maintenance endocrine/hormonal therapy will be included. Those with additional primary tumor diagnoses or primary chronic diseases will be excluded.

#### Interventions (I)

This review will include studies assessing BOIs, including psychomotor therapy, body awareness therapies, movement-oriented therapies, dance therapy, physical activity, tai chi, qigong, yoga, pilates, mindfulness, meditation, relaxation, and guided imagery.

#### Comparators (C)

Studies must include a control group receiving either no intervention, standard care, or a non-BOI.

#### Outcomes (O)

The primary outcomes of interest include preintervention and postintervention measures of cancer- and treatment-related symptoms, including fatigue, pain, sleep quality, psychological distress, body image, well-being, and QoL. Measurements of the above outcomes may be obtained by self-reported measures or researcher-administered measures.

#### Study Design (S)

Randomized controlled trials (RCTs), quasi-RCTs, and pilot studies will be included.

### Search Strategy

Two independent researchers will conduct searches in the following electronic bibliographic databases: PubMed, Cochrane, Web of Science, Scopus, APA PsycNet, and Portal Regional da BVS. Searches will be performed using the title, abstract, and keyword fields in each database. Search terms will include “breast cancer survivors,” “women who survived breast cancer,” “cancer-related symptoms,” “treatment-related symptoms,” “body-oriented interventions,” and “mind-body interventions.” Boolean operators “AND” and “OR” will be used to combine and refine these terms (Multimedia Appendix 2).

For each concept block, we will combine Medical Subject Headings (MeSH) with relevant free-text keywords. Database searches will be supplemented by reviewing grey literature sources (eg, theses, conference proceedings) and by manually screening the reference lists of all included studies to identify additional eligible reports.

### Study Selection

Two reviewers will independently select relevant literature according to the PRISMA flowchart (Figure 1) and predefined eligibility criteria.

After completing the database searches, the retrieved articles will be compiled in EndNote 20 reference manager [26] to facilitate title and abstract screening and remove duplicates.

In the initial selection process, the two reviewers will assess the titles and abstracts, screening them against the predefined inclusion criteria. Articles that do not meet the criteria will be excluded. In cases where the two reviewers disagree, a third reviewer will be consulted to resolve discrepancies.

For articles deemed potentially relevant, full-text articles will be reviewed to confirm eligibility based on inclusion and exclusion criteria. A third reviewer will resolve disagreements.

All members of the review team are native Portuguese speakers with fluent proficiency in English, French, and Spanish, and have extensive experience in the critical appraisal and interpretation of scientific literature. Titles, abstracts, and full texts will be screened independently by two reviewers; any uncertainties in translation or interpretation will be discussed within the author group until consensus is reached. Where ambiguities persist, we will consult additional native speakers or contact study authors for clarification. This approach ensures both linguistic accuracy and methodological consistency across all included studies.

### Data Extraction

Two reviewers will independently extract, via a standardized form, the following from each included study: (1) study details (author, year, country, design, sample size); (2) participant characteristics (age, time since treatment); (3) intervention and comparator specifics (BOI modality, dose/intensity, delivery format, control description); (4) outcomes and results (measures for cancer-related symptoms, well-being and QoL, with effect estimates, variance and time points); and (5) methodological quality and context (risk‐of‐bias ratings, adverse events). Discrepancies will be resolved by discussion or third-reviewer adjudication.

In our systematic review, we will also perform the following:

- Group studies by BOI modality, for example, psychomotricity, dance/movement, relaxation techniques, structured exercise.
- Within each modality, organize and summarize findings across 3 predefined outcome domains: (1) cancer- and treatment-related symptoms (eg, fatigue, pain, sleep quality, menopausal symptoms, lymphedema, body-image alterations); (2) well-being (eg, global and domain-specific well-being measures); and (3) QoL (global and domain-specific QoL measures).
- Apply a best-evidence approach by identifying the highest quality studies, summarizing their key effect estimates and methodological rigor, and grading the body of evidence (risk of bias, consistency, directness).

This framework provides a transparent, a priori structure, focused on intervention type and outcome domain, while preserving the flexibility needed to adapt to whatever the literature yields.

### Quality Assessment

The PEDro Scale [27] will be used to evaluate the methodological quality of the included studies and to evaluate the risk of bias [28]. This scale evaluates the internal validity and the interpretability of trial outcomes based on 11 items, of which 10 contribute to the total score; so, a sum score ranging from 0 to 10 can be obtained, with higher scores indicating better methodological quality, and according to Moseley et al [29], a score of <5 indicates “low quality RCT” and a score ≥5 indicates “high quality RCT.” The first item, which is not included in the total score, relates to external validity. The PEDro scale ensures that trials meet essential criteria for methodological rigor, reducing bias and improving the reliability of the findings [30]. Two reviewers will independently score the methodological quality of each study, and a third reviewer will arbitrate discrepancies. While the PEDro scale will be used to assess the methodological quality of included studies, we acknowledge that it does not fully cover domains such as selective outcome reporting and other sources of bias. Therefore, to ensure a more comprehensive assessment of internal validity, we will also apply version 2 of the Cochrane Risk of Bias tool (RoB 2) [31]. In cases where PEDro and RoB 2 assessments differ, priority will be given to RoB 2 for risk-of-bias judgment, given its domain-based and outcome-specific approach. PEDro scores will be used to provide a standardized measure of methodological quality, particularly for physical- and movement-related interventions, and will complement RoB 2 findings by offering a numerical summary of trial design rigor. Both assessments will be reported, and any major discrepancies will be discussed in the narrative synthesis to ensure transparency.

### Best Evidence Synthesis

The Best Evidence Synthesis [32] will be used to determine the strength of the scientific evidence. Best Evidence Synthesis serves as an alternative to meta-analysis, focusing on applying rigorous and well-founded criteria to extract unbiased and meaningful insights from experimental studies [32]. Based on the methodological rigor and consistency of findings, studies will be classified as providing strong evidence (derived from multiple high-quality RCTs), moderate evidence (from one high-quality RCT combined with one or more low-quality RCTs), limited evidence (from one high-quality RCT or several low-quality RCTs), and no evidence (from a single low-quality RCT or studies with conflicting results) [33]. To address expected heterogeneity, studies will be grouped by type of BOI (psychomotricity, dance/movement, relaxation techniques, structured exercise) and outcome domain (cancer- and treatment-related symptoms, well-being, or QoL). The primary analysis and strength of evidence grading will focus on outcomes measured immediately post-intervention. Other follow-up periods (short-term, long-term) will be described narratively. All eligible studies will be included in the synthesis and assessed for methodological quality by using tools such as the PEDro scale and RoB 2. While all studies will contribute to the narrative synthesis, the overall strength of evidence grading will give greater weight to studies with low to moderate risk of bias, whereas findings from studies with high risk of bias will be interpreted with caution.

## Results

This protocol describes the prespecified methodology for the systematic review. Although the corresponding full systematic review manuscript has been under review for publication at the time of publication of this protocol, the review methods were defined a priori. The outcomes will synthesize the scientific evidence on the effects of BOIs on cancer- and treatment-related symptoms in WSBC. It is anticipated that this systematic review will identify benefits and directions for future research to support the integration of BOIs tailored to this population.

## Discussion

This protocol outlines a systematic review designed to elucidate the impact of BOIs on cancer-related symptoms, well-being, and QoL in WSBC. Simultaneously, the review will identify evidence gaps such as understudied modalities, inconsistent outcome measures, and methodological limitations to inform future research and support the tailored integration of BOIs into survivorship care.

This systematic review will synthesize the scientific evidence on the effects of BOIs on cancer- and treatment-related symptoms in WSBC. It is expected that this systematic review will identify the benefits and directions for future research to support the integration of BOIs tailored to this population.

The systematic review may help clarify whether body and movement awareness contributes to the therapeutic effects of BOIs in cancer rehabilitation. By synthesizing evidence across diverse BOI modalities, we will underscore the role of embodied self-regulation, clarifying whether interventions that increase survivors’ attunement to interoceptive signals of tension, fatigue, or pain generate feedback loops that support adaptive coping and stress modulation [34,35], thereby improving health, well-being, and QoL. Additionally, the review will anchor these findings within embodiment frameworks—which posit that health emerges from the dynamic interplay of bodily action and psychological state [36], positioning BOI as a mechanistic bridge between somatic regulation and cognitive-affective restoration [37]. Taken together, this theoretical lens will highlight body-and-movement awareness/regulation not as ancillary “add-on” components but as foundational processes through which BOIs exert their therapeutic effects in breast cancer survivorship.

Through our best-evidence narrative synthesis, this review may provide practical guidance for clinicians on integrating BOIs into breast cancer survivorship care. By examining intervention dose and delivery for all the identified BOIs, we will specify the frequency, duration, and format (individual versus group) most consistently associated with meaningful reductions in cancer- and treatment-related symptoms, well-being, and QoL. We will then match these parameters to specific BOIs (eg, recommending dance and movement therapies to address body-image concerns or relaxation-based techniques to improve sleep quality), thereby enabling targeted intervention selection. We may also be able to propose models for interdisciplinary collaboration, outlining pathways by which psychomotor therapists, physiotherapists, exercise physiologists, psychologists, oncologists, and other psycho-oncology professionals can work together to prescribe and/or deliver BOIs seamlessly within existing care structures. Collectively, these insights will empower evidence-based rehabilitation prescription, sharpen clinical decision-making, and underpin the development of practice guidelines tailored to the unique needs of WSBC.

A key strength of this review is the inclusive scope, encompassing a wide range of BOI modalities and cancer- and treatment-related symptoms, unlike prior reviews with narrower focus [10,11,38]. Considering previous systematic reviews focused on the effects of BOIs in survivors of all cancer types [16,17], we anticipate challenges related to heterogeneity in intervention approaches, study designs, and subjective outcome measures (eg, QoL and body image), which may introduce some inconsistency and imprecision. To mitigate these issues, we will follow PRISMA guidelines and conduct detailed assessment of risk-of bias, methodological quality, and strength of evidence.

In conclusion, this review will provide reliable, high-quality evidence to inform health care professionals and future research, ultimately improving survivorship care for WSBC.

## Appendix

Multimedia Appendix 1 PRISMA-P-checklist for systematic reviews protocols.

Multimedia Appendix 2 Detailed search strategy.

## Abbreviations

BOI: body-oriented intervention
MeSH: Medical Subject Headings
PICOS: population, intervention, comparison, outcome, and study design
PRISMA-P: Preferred Reporting Items for Systematic Reviews and Meta-analyses Protocols
PRISMA: Preferred Reporting Items for Systematic Reviews and Meta-Analyses
QoL: quality of life
RCT: randomized controlled trial
RoB 2: version 2 of the Cochrane Risk of Bias tool
WSBC: women who survived breast cancer
