# Supplementary material for: The Effects of Body-Oriented Interventions on Cancer-Related Symptoms of Women Who Survived Breast Cancer: Protocol for a Systematic Review
Source: JMIR Res Protoc. 2026 May 1;15:e76858. doi: 10.2196/76858 (PMC13179483; doi:10.2196/76858)
Supplement: Multimedia Appendix 2 [file resprot_v15i1e76858_app2.docx]

Search Strategy:

((((((breast cancer survivor* OR survivors of breast cancer* OR women after breast cancer* OR breast cancer* OR breast carcinoma* OR breast neoplasm* OR breast tumor*) AND (physical health* OR mental health* OR quality of life* OR health* OR well-being* OR fatigue* OR physical function* OR pain* OR inflammatory processes* OR stress* Or sleep quality* OR depression* OR anxiety* OR social relationship* OR body image* OR body schema* OR body satisfaction* OR self-esteem* OR self-concept* OR cancer-related symptom* OR treatment-related symptom* OR psychological symptom* OR physiologic symptom*) AND (psychomot* OR body-oriented* OR mind-body* OR movement oriented* OR dance and movement* OR danc* OR body awareness* OR body psychotherap* OR psychomotor physiotherap* OR exercise* OR physical activit* OR sport* OR run* OR tai-chi* OR qigong* OR baduanjin* OR yoga* OR pilates* OR mindful* OR meditation* OR relaxation* OR stretching exercises* OR guided imagery* OR recreational* OR dram*) AND (therap* OR intervention* OR program* OR approach*) AND (randomized OR trial OR RCT OR experiment*) NOT (systematic review* OR literature review* OR male* OR child*)

Example:

| Database | Strings | Filters |
| --- | --- | --- |
| Pubmed | ((((((breast cancer survivor* OR survivors of breast cancer* OR women after breast cancer* OR breast cancer* OR breast carcinoma* OR breast neoplasm* OR breast tumor*)) AND ((physical health* OR mental health* OR quality of life* OR health* OR well-being* OR fatigue* OR physical function* OR pain* OR inflammatory processes* OR stress* Or sleep quality* OR depression* OR anxiety* OR social relationship* OR body image* OR body schema* OR body satisfaction* OR self-esteem* OR self-concept* OR cancer-related symptom* OR treatment-related symptom* OR psychological symptom* OR physiologic symptom*))) AND ((psychomot* OR body-oriented* OR mind-body* OR movement oriented* OR dance and movement* OR danc* OR body awareness* OR body psychotherap* OR psychomotor physiotherap* OR exercise* OR physical activit* OR sport* OR run* OR tai-chi* OR qigong* OR baduanjin* OR yoga* OR pilates* OR mindful* OR meditation* OR relaxation* OR stretching exercises* OR guided imagery* OR recreational* OR dram*)))) AND ((therap* OR intervention* OR program* OR approach*))) AND ((randomized OR trial OR RCT OR experiment*))) NOT ((systematic review* OR literature review* OR male* OR child*)) | *Randomized controlled trial; Clinical trial;*  *Female;*  *Adults 19+;*  *Young Adult 18-24;*  *Adult 19-44;*  *Middle aged + aged 45+;*  *Middle aged 45-64*  Language: english, french, portuguese, spanish |

This search strategy combines free-text terms and truncations. Where applicable, equivalent MeSH terms will be included or mapped in each database (e.g., PubMed).
